# Perceptions of neuromuscular electrostimulation of patients with Parkinson’s disease and their caregivers: the impact on swallowing and speech from a qualitative perspective

**DOI:** 10.1186/s41687-025-00931-z

**Published:** 2025-10-17

**Authors:** Mariana de Almeida Simão, Silvia Poveda-Moral, Elizabeth Ramírez Daniel, Aracely Domínguez Ganora, Militza Vergara Vargas, Jaime Kulisevsky

**Affiliations:** 1 Department of Medicine, Universitat Autònoma de Barcelona, Barcelona, Spain; 2 Study Location and Focus Groups, Ethics Committee, Sant Antoni Maria Claret 167, Barcelona, 08025 Spain; 3 Faculty of Nursing, Universitat de Barcelona, Calle de la Feixa Llarga s/n, L’Hospitalet de Llobregat, Barcelona, 08907 Spain; 4 Atos Medical Spain, Barcelona, Spain

**Keywords:** Quality of life, Health-related quality of life, Dysphagia, Dysarthria, Parkinson's disease, Neuromuscular electrostimulation

## Abstract

**Purpose:**

Parkinson’s disease (PD) is a progressive neurodegenerative disorder that manifests itself through motor and non-motor symptoms, which significantly influence quality of life. We aimed to explore the experiences of patients with PD and their caregivers before, during and after receiving treatment with neuromuscular electrostimulation (NMES), and to analyse how they perceive the changes experienced, especially in relation to swallowing and speech.

**Methodology:**

A phenomenological qualitative study was conducted through 11 focus groups (FGs): 7 with patients and 4 with caregivers, held separately. Group sizes ranged from two to six participants. The decision to include small groups, including those with only two participants, was made to achieve theoretical saturation of the data. A manual inductive thematic analysis of discourse was performed, coding responses into emergent categories.

**Results:**

The study sample consisted of 38 participants: 24 patients and 14 caregivers. The results fell into two broad categories: patient experience and carer experience. In terms of patients’ experiences, the majority reported significant improvements in swallowing, salivation and, to a lesser extent, speech. Regarding caregivers, the improvements observed in patients were perceived as a relief in their daily caregiving responsibilities, facilitating meal preparation, enhancing communication, and providing greater reassurance in supporting the treatment process.

**Conclusion:**

This qualitative study provides valuable insights into NMES treatment in PD, showing improvements in areas such as swallowing, speech and saliva. By including the experiences of patients and carers, we identify critical aspects that impact on daily life that cannot be fully explored by other methods. The findings highlight the importance of a more holistic and individualised approach to treatment. It is suggested that future qualitative research should continue to explore these experiences with a view to improving rehabilitation strategies and health-related quality of life (HRQoL) for people with PD.

## Abstract (plain language summary)

This study is relevant because Parkinson’s disease seriously affects people’s daily lives, including aspects such as swallowing and speech. We wanted to better understand how patients felt after receiving neuromuscular electrostimulation (NMES) treatment, and their caregivers’ perceptions of this treatment; this information could help with these difficulties. Thus, we conducted focus groups to explore the experiences and perceptions of patients with Parkinson’s disease and their caregivers. Some patients noted that they were able to swallow better, to control and produce saliva more easily, and to take their medication more easily; however, most did not report improvements in speech. Caregivers, both formal and informal, also noted these improvements in patients. They were motivated to support continued treatment based on the positive changes they observed in the daily lives of the people for whom they provided care. In conclusion, this study suggests that, in the experience of the participants, NMES may have beneficial effects on some of the difficulties faced by people with Parkinson’s disease in their daily lives.

## Introduction

Parkinson’s disease (PD) is a chronic and progressive neurodegenerative disorder that affects both the motor function and quality of life (QoL) of patients [1, 2]. Among motor symptoms, oropharyngeal dysphagia (OD) is particularly relevant, as it compromises swallowing and is associated with significant impairment of health-related quality of life (HRQoL) [2, 3]. Another fairly common motor symptom is hypokinetic dysarthria, which is characterised by difficulty in articulating and monotonous speech, which negatively affect a patient’s ability to communicate [4]. Speech therapy intervention, focusing on speech and swallowing, is crucial to mitigate these effects and to improve HRQoL [2, 3, 5].

Although conventional speech therapy is essential, it is not always sufficient to treat dysphagia and dysarthria in patients with PD. Neuromuscular electrical stimulation (NMES), which is approved by the FDA for the treatment of dysphagia, can be used to stimulate motor neurons in orofacial muscles and swallowing. In PD, NMES may modulate neuromuscular function through two primary mechanisms: (1) the facilitation of neuroplasticity in corticobulbar circuits, and (2) reduction of the characteristic muscular hypoactivation in this pathology. However, optimal application protocols (intensity, frequency and duration) still require further scientific evidence [6–8].

It is also important to consider the impact of treatment on caregivers, who bear a significant burden when caring for people with PD. Caregiver HRQoL is often compromised by PD progression, particularly when managing dysphagia-related complications (e.g. meal preparation time, fear of choking). As NMES may alleviate some symptoms, its potential to reduce caregiver burden – by improving patients’ autonomy in eating and communication – warrants exploration [9]. Although there are studies on HRQoL related to dysphagia and dysarthria in PD, most have focused on quantitative approaches; hence, there is a gap in our understanding of the lived experiences of patients and caregivers and their impact on QoL [2, 9–14]. In the present study, rather than directly measuring QoL, we set out to explore the experiences of patients with PD after they had received NMES treatment, and the perceptions of this treatment of their primary caregivers. We aimed to better understand how NMES treatment affects the overall well-being of patients with PD, with a particular focus on its influence on speech and swallowing.

Methodology.

### Type and design of study

This qualitative phenomenological study was conducted in 2022 in (Escola Clínica de Neuropsicologia i Patologia del Llenguatge del Hospital de la Santa Creu i Sant Pau). According to Edmund Husserl, this type of study aims to explain how individuals interpret and make sense of social phenomena through their own experiences [11, 15, 16]. This methodology was selected because it is the most appropriate to achieve the stated objective, which focuses on exploring, describing and understanding people’s experiences of a phenomenon and discovering the commonalities of these experiences through focus groups (FGs) [16]. Each FG is moderated by a person who leads the participants, so an FG represents a semi-structured and guided interview that seeks the group’s answers to questions [11].

### Ethical considerations

The study was conducted in accordance with the recommendations of the Declaration of Helsinki and the Belmont Report on human studies. In addition, approval was obtained from the Clinical Research Ethics Committee: IIBSP-EIO-2018-48 of the (Hospital de la Santa Creu iSant Pau). All subjects were informed, both verbally and in writing, about the voluntary nature of their participation, as well as the aims and procedures of the study. Signed informed consent was obtained from each participant, and a copy was given to all of them.

### Population and sample

The study was conducted in two phases. In phase 1, all participants – people with PD and their carers – received lingual NMES, supplemented by conventional speech therapy. NMES sessions were given twice a week for 30 min each for one and a half months, for a total of 12 sessions. This phase was developed through direct intervention with patients, always accompanied by their carers.

After the completion of phase 1, the qualitative phase of the study began, in which focus groups were organised separately: seven patient focus groups (PFGs) and four carer focus groups (CFGs). This design aimed to avoid power dynamics between patients and carers and thus encourage free and honest expression of lived experiences. The separation allowed for a comparison of independent perspectives between the two profiles involved in the intervention.

Purposive sampling was carried out by including patients and carers who had direct experience of the intervention developed in phase 1 of the study. In the case of carers, both formal and informal carers were included as inclusion criteria in order not to exclude any profile that had accompanied the patients during the process. In practice, however, all caregivers who agreed to participate in the FGs turned out to be informal caregivers.

The total sample for the qualitative study consisted of 38 participants: 24 PD patients (9 women and 15 men) and 14 informal carers (10 women and 4 men). Invitations to participate in the FGs were made through direct contact and telephone communication with participants who had completed phase 1 of the treatment.

Some patients and carers who participated in phase 1 of the intervention decided not to take part in the FGs corresponding to the qualitative phase of the study. In total, 21 people decided not to participate in the FGs (6 patients and 15 carers). As a result, although in some cases the participation of patients and their respective carers (dyads) was achieved, it was not possible to form a complete dyad for each participant. In other words, the simultaneous presence of the patient and their carer was not possible in all cases. The impossibility of forming complete dyads in all cases was due to various reasons. The most common reasons were incompatibility of days and times, and others not feeling comfortable participating in group activities. A detailed record of the individual reasons was not kept for each case, but no refusals due to disagreement with the study or the subject matter were recorded.

In this research, focus groups composed of two participants were included. Although this number is below the usual range for this technique, their inclusion was based on two fundamental methodological reasons. First, the preliminary analysis of previous groups showed that some dimensions of the phenomenon had not been sufficiently explored. Incorporating these groups allowed for a deeper exploration of these areas, which was key to achieving theoretical saturation of the data [17]. Second, the literature recognizes the validity of using mini focus groups as a methodological strategy in qualitative studies, especially when working with specific populations or requiring a more intimate setting to facilitate individual expression [18, 19]. Moreover, other studies indicate that saturation in FG can be reached between four and eight sessions, depending on participant homogeneity and study objectives, so conducting smaller groups can be useful to consolidate and finalize thematic analysis [17, 20].

For identification and confidentiality purposes, an internal alphanumeric coding system was used for FG participants. For example, the first FG with patients was coded as GFP1, GFP2, and so on, while if the next group was with carers, the correlative numbering continued, using the corresponding abbreviation GFC to differentiate them, followed by the number assigned (GFC3, GFC4, and so on). This system made it possible to guarantee the confidentiality of the participants and to organise the data in a structured way.

### Data collection instruments

There were two main data collection instruments for this study. The first was a protocol of procedures and tasks that was adopted before the arrival of the participants and throughout the recording of the FGs. The procedures were related to the set-up of the room, the materials and how to audio-record the sessions on a computer. Second, there were two semi-structured scripts – one with 12 questions for patients with PD and one with 10 questions for caregivers – to find out about their experiences during and after the speech intervention process using NMES. The interview guides were developed by the research team based on clinical scripts used in previous studies – such as the Swallowing Disturbance Questionnaire [21] and the Swallowing Quality of Life Questionnaire [22] – which were adapted and contextualised according to the specific objectives of this study. In addition, the guides were reviewed by two expert researchers — one specialising in oropharyngeal dysphagia and the other in qualitative methodology — to ensure clinical relevance and consistency with the study objectives. The (withdrawn for review) provided a room for each FG on different days. An Apple^®^ MacBook Pro computer with QuickTime Player software was used to audio-record each FG. Sonix software was then used to automatically transcribe the audio recordings. However, after an initial review, errors were identified in the automatically generated transcripts. A two-stage manual verification process was therefore implemented to ensure the validity and fidelity of the transcripts to the original audio. First, each transcript was fully reviewed and corrected by one of the researchers. A second researcher then rechecked all material to ensure the consistency and accuracy of the transcribed data. This double check ensured the quality of the transcripts before proceeding with the data analysis.

### Data collection and analysis procedures

Each FG was conducted by following a specific protocol that outlined the necessary procedures and tasks. In addition, socio-demographic data were collected for both patients and caregivers to understand the profile of each participant and their family context. The computer used to audio-record the FG was positioned on a table close to where the participants were sitting but facing away from them so that they would not be distracted by it or have feedback from the recording, a factor that could affect their performance. After establishing the recording conditions, discussion was facilitated using the semi-structured scripted questions for both patients and caregivers. It should be noted that the topics explored were defined and agreed upon in advance by the research team, and that Table 1 presents a representative selection of the questions included in the semi-structured guides, with the purpose of illustrating the thematic areas addressed during the FG, without reflecting the full set of questions posed.

**Table 1:** Guiding questions for patients and their carers

Study area	Questions
**Patients**	
Changes observed with neuromuscular electrostimulation treatment	How would you describe the impact that doing this treatment has had on your life?
Neuromuscular electrostimulation treatment logistics	What did you think of the frequency with which the sessions were held? What changes or improvements would you make to the sessions and/or intervention?
Changes in diet	What has your experience been like swallowing the food/medicines you are used to taking after electrostimulation treatment?
Speech changes	What changes have you noticed in your speech after electrostimulation treatment?
Socialization	Describe how it feels to eat in the presence of company or in public places.
**Carers**	
Changes in the feeding of your relative/patient	What benefits did you observe, in your family member/patient’s daily routines, in relation to food?
Barriers and facilitators as a carer	What barriers do you experience as a caregiver during this treatment?
Changes in relation to the treatment of neuromuscular electrostimulation in the family member/patient	How did you observe the patient during treatment? According to your experience as a caregiver, do you consider that the electrostimulation treatment achieved any change in the quality of life of your relative/patient?
Changes in the feeding of your relative/patient	What benefits did you observe, in your family member/patient’s daily routines, in relation to speech?

The 11 FGs – encompassing seven patient-only FGs andfour caregiver-only FGs – consisted of a minimum of two and a maximum of six participants. To guarantee the confidentiality of the information, each participant was assigned a code. This information is exclusively accessible to the research team and is in strict accordance with the data protection laws currently in force (Law 41/2002, Organic Law 3/2018 and the European Personal Data Protection Regulation). All FGs were moderated by the research team (one leader and two observers). There was no professional or personal relationship between the moderators and the participants. The duration of each FG varied according to the number of participants, ranging from 40 to 90 min. After each FG, the audio files were exported and transcribed.

We conducted a thematic discursive analysis following the six-stage method proposed by Braun and Clarke [23]. This analysis was carried out through a manual inductive process in which the research team followed all the successive phases in order to ensure a detailed and coherent interpretation of the collected material. In the first phase, a comprehensive exploratory reading of all FG transcripts was carried out in order to become familiar with the content and begin to identify general ideas. During this phase, first impressions were also noted down and a search for structures and meanings in all available information was started. In the second phase, relevant units of meaning were identified. These units included salient phrases, frequent expressions, emotions expressed or reflections shared by the participants. Preliminary codes were assigned to each unit, allowing the data to be organised from the outset. In phase 3, the preliminary codes were grouped into thematic categories. These categories were constructed based on conceptual similarities and emerging patterns in the participants’ discourses. As codes were grouped, they were assigned a level of abstraction corresponding to their meaning and relevance. In phase 4, the thematic categories were reviewed and refined by looking for relationships between them and reorganising the codes where necessary. This step made it possible to progress to phase 5: establishment of the category hierarchy. In this step, the progressive category hierarchy of first- and second-level codes was defined, depending on the level of abstraction and the complexity of the content. The categories were hierarchised to ensure that the final structure reflected the most significant relationships and connections between the identified themes. Finally, in phase 6 – final organisation and presentation of findings – two tables were created to organise the information collected from patients and caregivers. These tables were key to facilitating the understanding and interpretation of the data. The organisation of the information allowed the findings of the qualitative analysis to be presented in a clear and structured way. This process of analysis was developed collaboratively among the researchers, with regular debriefing and discussion sessions to ensure interpretative coherence and depth of analysis.

### Quality and rigour criteria

To ensure the quality and rigour of this research, the criteria proposed in 1985 by Guba and Lincoln (credibility, transferability, dependability and confirmability) were adopted.

With regard to credibility, we employed triangulation to maintain scientific rigour. The FGs interviews were conducted by three researchers, who also independently coded the transcripts, which allowed us to contrast and enrich the findings from different professional perspectives. All three researchers were clinical speech and language therapists with a master’s degree in oropharyngeal dysphagia and experience in neurological dysphagia and had been trained by an expert in qualitative methodology who participated in the entire data analysis process. Consensus meetings were then held to discuss and refine the emerging categories. A fourth researcher, a nurse with a PhD in health sciences, specialised in qualitative methodology, attended these sessions and provided a complementary methodological view. Any critical discrepancies were resolved through internal refereeing led by the doctor, drawing on her experience of qualitative methodological work. In addition, the findings were also validated by the participants. As regards transferability, a detailed explanation of the context and profile of the participants was provided, which may help other researchers to replicate this same study in similar contexts. Finally, and in reference to dependability and confirmability, a detailed record of the entire methodological process and a process diary were kept.

This process ensured the coherence and interpretative depth of the findings, following the criteria set out in the Consolidated Criteria for Reporting Qualitative Research (COREQ) tool [24].

## Results

### Patients

The study sample consisted of 38 participants: 24 patients and 14 caregivers. The 24 patients were all of Spanish nationality; nine were female and 15 were male, and their ages ranged from 60 to 87 years. In terms of the family environment, all of the patients’ carers are immediate family members. The majority are their spouses, with the exception of three cases: two patients are cared for by their children and one by a combination of son and sister. One patient has no spouse or carer and lives alone, which is a unique situation within the group.

Seven FGs were held with patients: PFG1 comprised five patients, PFG2 four, PFG4 two, PFG6 six, PFG8 two, PFG10 two and PFG11 included three patients. Based on a manual inductive analysis, the content was organised into the categories and codes shown in Table 2 and in the section on patients’ experiences. Five first-level categories were identified. Each category is described below. When quotes are presented, the participant is identified by the FG number and patient code (e.g. PFG2_P8 refers to patient 8 from PFG2).

**Table 2:** Categories and codes from the patient focus groups

Second-level category	First-level category	Codes
Patient experiences	Experiences with neuromuscular electrostimulation	Supportable
		No discomfort
		Well tolerated
		Fear at the beginning, it was strange
		The changes are maintained
		Novel
	Impact on swallowing	Better swallowing of pills
		Eating and drinking better
		Eating faster
		More strength on the tongue
		Chewed faster
		I haven’t noticed anything, everything is the same
	Impact on speech	Unchanged
		They understand me more but I don’t realize it
		Improved, I speak more clearly
	Impact on oral sensation	I got better with saliva, I don’t have so much dryness
		No impact
		I don’t choke on saliva
		Increased saliva
		Decreased saliva
		It has improved more when I chew
	Impact on social relations	I feel uncomfortable eating with others, I avoid it
		I don’t feel uncomfortable, but I finish last and change consistencies
		As slower
		I have no problems
	Treatment logistics	Good frequency
		If it seemed right to me
		Very short treatment
		More sessions, more exercises
		It made me feel better
		I would recommend it
		Positive effects
		Better once a week
		Two or three days a week would be good
		I would do it again
		Have a guideline with exercises
		Not a study, but a form of rehabilitation.
		No, I would not advise it because the effects can be different from person to person

### Experiences of NMES treatment

The experience of NMES treatment was perceived by most patients as novel and non-invasive. Despite some initial scepticism or uncertainty, all patients described the intervention as tolerable, innovative, pain-free and without adverse effects. In addition to good acceptance, participants reported perceived positive effects on swallowing-related functions, such as enhanced intraoral sensitivity, and improvements in bolus control and muscle strength involved in chewing. Also, albeit to a lesser extent, some reported a perceived improvement in speech articulation.

*[…] No*,* I felt fine*,* a bit strange because I had never felt electrostimulation on my tongue […]* (PFG 2_P8; PFG4_P14).

*[…] When the intensity increased*,* it was felt a little*,* but it was bearable*,* without pain*,* well tolerated […]* (PFG2_P7).

*[…] Eating better and it is maintained […]* (PFG1_P5).

*[…] They still don’t bite me*,* that’s why it stays the same […]* (PFG2_P7).

However, two participants showed no clinical changes attributable to NMES, although they tolerated it well.

*[…] No discomfort*,* no pain*,* but this did not allow me to see any difference […]* (PFG1_P3).

*[…] I was quite curious*,* it didn’t bother me*,* it tingled*,* but I didn’t feel any changes […]* (PFG11_P38).

### Changes in swallowing

A significant proportion of patients reported improvements in swallowing, particularly when swallowing medications. Some also reported improvements in preparing and swallowing solid food. These patients said that they were able to swallow food and medicines more efficiently, without the discomfort or difficulty they had previously experienced.

*[…] I improved my intake of medication*,* I feel that I am able to bring my tongue to the roof of my mouth better […]* (PFG1_P5; PFG1_P4).

*[…] I didn’t notice it in the medication*,* I noticed it in the food […].* (PFG2_P7; PFG2_P8)

*[…] Yes*,* it is better for chewing food*,* my mouth feels wetter and I can swallow better […].* (PFG4_P14; PFG2_P7)

However, not all patients experienced these benefits. Some did not notice any significant changes in their swallowing, either during or after treatment. Two of them mentioned the following:

*[…] I have no changes*,* neither during nor after treatment […]* (PFG11_P37).

*[…] I don’t feel so many problems*,* I didn’t see any improvement […]* (PFG11_P38).

### Speech disturbances

In general, participants did not notice any changes in their speech during or after treatment. One even reported increased difficulty in articulation. Although no objective improvements were reported in general, speech emerged as a particularly sensitive aspect of patients’ discourse. During the focus groups, the emotional charge associated with communication difficulties was evident: many expressed feelings of frustration, sadness or embarrassment when discussing this topic, and some became visibly emotional. Speech impairments affected not only their personal well-being but also their social relationships, both with family members and strangers.

*[…] Exactly the same as before*,* now the voice is lower […]* (PFG1_P2).

*[…] I haven’t noticed any difference […]* (PFG1_P3; PFG11_P36).

In contrast, two participants reported a perceived improvement in their speech, supported by comments from people close to them that they spoke more clearly or fluently.

*[…] My speech has improved; I am told by acquaintances that I speak more clearly […]* (PFG6_P22; PFG4_P14).

### Oral sensations

The majority of patients interviewed reported changes in saliva production or control after treatment. Many reported an increase in salivation, which was viewed positively as contributing to easier chewing and bolus formation. In contrast, some patients described a decrease in salivation associated with improved oral control, particularly at rest or when speaking. These perceptions were reported both during and after treatment and were associated with an enhanced sense of well-being.

*[…] Saliva increased after the treatment*,* I don’t have the sensation of a dry ball in my mouth with food […]* (PFG1_P2).

*[…] I improved my saliva*,* it caused me discomfort when I ate*,* now I have more sensitivity and it remains to this day […]* (PFG4_P15).

*[…] What I do think I have improved is the saliva drop*,* I have less salivation than before […]* (PFG11_P36).

Other participants reported no change in this area.

*[…] The saliva leakage is the same as before the treatment and there has been no change*,* the only thing is that I don’t choke on the saliva […]* (PFG1_P2).

*[…] I continue with a lot of dryness […]* (PFG10_P33).

### Social consequences associated with eating

Most patients reported no significant difficulties in their social lives related to eating and generally continued their social routines with friends and family without major inconvenience.

*[…] I have no shame*,* since I got Parkinson’s I go out a lot […]* (PFG1_P3; PFG1_P2; PFG11_P36).

*[…] I have no problem eating in company or in public places […]* (PFG1_P4).

*[…] I haven’t noticed it*,* I don’t have any problems*,* I don’t care […]* (PFG2_P8).

However, one group of participants reported some impact on their social interactions, particularly during mealtimes. These patients expressed discomfort when eating in the presence of others, either due to eating more slowly or because of experiencing problems such as dropping food. Some also mentioned that, in social contexts, they chose softer foods to make chewing and swallowing easier, and did not prolong mealtimes excessively, reflecting an adaptive strategy to cope with perceived ingestive difficulties.

*[…] When I eat I mash the food more so I don’t cough*,* I prefer to eat alone rather than with company […]* (PFG1_P5).

*[…] I feel a bit bad eating with others because I drop the food […]* (PFG2_P6; PFG4_P14).

*[…] I have to think about what I order when I go outside*,* I try to order something that is easy for me to eat […]* (PFG2_P9).

*[…] I have not stopped eating out but I do feel more uncomfortable because it is harder for me to prick my food […] and now I am slower and that also makes me uncomfortable […]* (PFG11_P37).

### Caregivers

All 14 caregivers were of Spanish nationality; 10 are women and four are men, and their ages range from 58 to 74 years old. All of the caregivers are married except for one, who is divorced. The vast majority of them have completed secondary school, some have a degree, and two of them have completed primary school. Regarding family environment, all patients’ carers were immediate family members. Most were spouses, except in three cases: two patients were cared for by their children, and one by a son and sister jointly. One patient had neither a spouse nor carer and lived alone - a unique situation within the cohort.

Four FGs were conducted with the patients: CFG3 with four carers, CFG5 with two, CFG7 with five and CFG9 with three. Based on the manual inductive analysis, the content was divided into categories and codes as shown in Table 3 and within the experiences with caregivers. Four first-level categories were identified. Each category is described below. When quotes are presented, the participant is indicated by the FG number and the caregiver code (e.g. CFG3_C10 indicates caregiver 10 from CFG3).

**Table 3:** Categories and codes based on the carer focus groups

Second-level category	First-level category	Codes
Experiences of carers	Emotional impact of their relatives	Motivated
		Content
		Proud to be able to help
		Strong will
		Exhausted but delighted with life
		Willing to cooperate
		Wants to solve the problem
	Carers’ perception of swallowing	To normal food
		You can eat better
		You can drink liquids better
		Improved after the study
		No need to grind food
		Feeling more confident when eating
		Can eat chunks
		I see improvement and it is maintained, although it still chokes but it is less repeated
		In food it has not changed in consistency
		No problems before or after
		I cut everything very small and it doesn’t have the slightest problem
	Perception of the cared-for person’s speech	More fluent speech
		Before she was upset because she was not understood, now she does not express it to me
		During the speech treatment he noticed an improvement in his speech
		No change in speech
		Sometimes people don’t understand him and realize that
	Changes in the saliva of the cared-for persons	Further change in saliva
		Dryness improved
		I used to salivate more than I do now
		He complained of dry mouth and now much less dry mouth
		Saliva improved and has been maintained
		Not much of a change but improved in saliva
	Carer’s experiences with neuromuscular electrostimulation treatment	No barriers, on the contrary
		We had to schedule ourselves around work schedules
		Informing the carer more and how to follow up at home
		I have to bring it, if the schedule is good, I have no problem
		She comes alone
		I would like this study to go on and on, because if they don’t, they will decline
		Give it a rest, see if it improves and then stimulate it again so that it does not lose
		Coming here he has not gone backwards, he has maintained himself and now he has tools

### Carers’ perceptions of treatment

The majority of carers reported that they perceived a mostly positive attitude towards treatment in their relative, highlighting emotions such as motivation, pride and a desire to improve. This active involvement during the sessions and the positive attitude towards the continuity of the therapeutic process led to feelings of satisfaction and relief among the carers.

*[…] He came motivated*,* happy and participative*,* proud to be able to help*,* you have to give them positive support […]* (CFG3_C10).

*[…] A lot of will*,* she was exhausted*,* but delighted […]* (CFG3_C11).

*[…] He was happy and motivated*,* he wants to solve the problem […]* (CFG5_C16).

However, three reports reflected different initial experiences. In these cases, carers described a more passive or reluctant attitude from family members at the beginning of the treatment, but with a positive development during the sessions. One carer noted that her relative did not want to participate and that it was necessary to play an active motivational role, relying on her prior knowledge of the process. She commented that this situation was particularly draining, noting that she found it exhausting to be constantly on top of her relative when she had her own health problems. In another testimony, the patient’s initial lack of motivation was attributed to a general mood rather than to the treatment itself. The carer reported that she felt emotionally affected by this situation, although she noticed significant improvements over time. She described the therapeutic process as beneficial not only for her relative but also for herself, as seeing his improvement made her feel better. Finally, in the third case, an initial attitude of scepticism on the part of the patient was mentioned, although as the sessions progressed he began to feel good and confident. The fact that he had completed the treatment was seen as an important achievement because, according to the carer, he had not accepted his condition, which was causing suffering for both of them. In her view, the process facilitated a progressive acceptance of both the diagnosis and his own limitations.

*[…] The treatment was great because he didn’t want to know anything*,* he would stay on the couch all day. At the beginning I had to motivate him […] I knew it would do him good*,* but it was very tiring*,* especially with my own health problems […]* (CGF9_C33).

*[…] At the beginning I saw him very discouraged to come*,* but it wasn’t because of the treatment*,* it was because of everything in general. But then he saw that he improved when he ate*,* he didn’t spit like before […] Seeing him like that makes me very happy. When he is well*,* so am I […]* (CGF7_C24).

*[…] On the contrary*,* it was very good*,* he came out of the treatment more open and accepting Parkinson’s in a different way*,* more open and with more awareness […]* (CGF3_C10).

### Eating with their family members

Carers were positive about the changes they saw in their family members’ swallowing after treatment. Many reported increased safety when eating both solid and liquid foods, with a significant reduction in the number of choking episodes. They also reported a reduction in the need to change the consistency of food, thus saving time when preparing meals. These improvements enabled some people to regain their previous eating habits. On the other hand, some carers did not observe any changes in feeding, while others mentioned adjustments in the utensils used, which they interpreted as a process of adaptation to new needs. These changes were perceived as facilitating the patient’s eating time and optimising the eating experience according to the carer’s observations.

*[…] I noticed that my wife used to complain about dry mouth many times when she ate and now she complains less […]* (CFG3_C13).

*[…] He improved after the treatment*,* before he choked a lot more with meals*,* it takes less time to eat […]* (CFG7_C27).

*[…] He eats with less sauce*,* he bites less […]* (CFG9_C31).

### Caregivers’ observations of their relatives’ speech

Several carers noted improvements in their relatives’ speech during and after treatment. Highlights included increased fluency, improved tongue mobility, and reduced dry mouth, which they observed as facilitating clearer articulation. Some carers mentioned that these improvements were particularly beneficial during conversations, as questions did not need to be repeated several times.

*[…] Improved tongue*,* more fluent speech […] Dry mouth improved and speech is more fluent […]* (CFG3_C13).

*[…] I had difficulties and I even noticed that during the treatment*,* afterwards I have seen that he doesn’t speak so monotonously […]* (CFG9_C32).

However, other carers did not see any significant changes in this area, and one carer even reported ongoing difficulties such as reduced voice volume or slowness of speech. It was also mentioned that it was often difficult to understand patients, which made communication difficult and in some cases led them to give up trying to continue the conversation.

*[…] His voice has dropped*,* he is not as fluent as before*,* he speaks more slowly and after the treatment there were no changes […]* (CFG3_C10).

### Changes in their family members’ saliva

Several carers reported improvements in saliva production during and after treatment. They mentioned a reduction in dry mouth, which made it easier to swallow food and to speak, making daily activities more comfortable. These changes were perceived as being sustained over time. However, one carer reported no change in this aspect, while others said they did not know if there was a relevant change in saliva production.

*[…] Saliva improved and has been maintained […]* (CFG5_C16).

*[…] Increased saliva during and after treatment […]* (CFG9_C32).

### Carers’ assessment of the treatment

All carers found the treatment beneficial. However, some mentioned that they had to rearrange their schedules in order to accompany their family members to the sessions. Most were motivated by the process and wished that more sessions were offered to help maintain the progress their family members had made. In addition, carers emphasised that the treatment not only benefited the patients but also allowed them to be actively involved, as attending the sessions enabled them to learn how to improve the care of their family members in their daily lives. Some carers commented that, beyond the trial, they saw value in incorporating NMES treatment as part of their regular speech and language therapy, including the opportunity to provide ongoing follow-up and support to ensure that patients do not lose the gains they have made, and to allow carers to contribute to the process from home.

*[…] On the contrary*,* it was very good*,* he came out of the treatment more open and accepting Parkinson’s in a different way*,* more open*,* he took the study as a medication that he has to take every day […]* (CFG3_C10).

*[…] I see improvement*,* it is true that I would like this study to never end because they can fall off*,* go back to the habit they had of eating quickly and choking […]* (CFG3_C11).

## Discussion

Our study has provided insight into the experiences of patients with PD and their primary caregivers during and after NMES treatment. According to the experiences reported by the participants, NMES treatment was perceived as beneficial by the majority, especially in terms of improvements in swallowing ability. The patients reported a reduction in the need to grind food and in the number of choking episodes. On the other hand, the caregivers reported improvements in food preparation, mentioning that they needed to adjust the consistency of meals less and use thickening agents less frequently in liquids, and they noticed a decrease in the number of choking episodes. These findings are in line with previous studies highlighting the benefits of NMES for dysphagia [6–8]. Despite these positive results, the literature shows considerable variability in the reported effects and suggests that the efficacy of NMES for swallowing in patients with PD is not fully established. This underlines the need for further research to confirm these effects and to better define the role of NMES in the treatment of dysphagia in this population [25, 26].

In addition to improvements in swallowing, the patients in our study reported positive experiences related to xerostomia and sialorrhoea. Specifically, several patients mentioned a decrease in dry mouth and improved food management during chewing and swallowing. Some who complained of hypersalivation also noted a reduction in salivation. These qualitative findings suggest that NMES may have a beneficial role in saliva management in patients with PD. This is particularly relevant given that PD affects salivation, manifesting as xerostomia due to reduced salivary flow and sialorrhoea due to facial and oropharyngeal hypokinesia [27]. Although these results are encouraging, given the variability in salivary symptoms (xerostomia/sialorrhoea), future studies should evaluate NMES protocols tailored to patients’ baseline dysfunction.

In regard to dysarthria, most patients reported no subjective improvement in speech, though some caregivers observed increased fluency. This discrepancy suggests that perceived changes may depend on the observer’s role and the communication demands – a finding in line with Clark’s [28] observation that NMES effects on dysarthria remain inconsistent across studies. Future research should incorporate both patient-reported outcomes and caregiver assessments to capture this variability.

Our study provides novel qualitative data by exploring the experiences of patients with PD and dysphagia following treatment with NMES. Although there have been quantitative studies on dysphagia and QoL [10, 11], our qualitative approach provides a deeper understanding of individual experiences. While the quantitative studies indicate general improvements, our qualitative findings suggest that patients face specific challenges that require detailed attention. Therefore, it is crucial to design specific therapeutic protocols that are based on the predominant symptoms of each patient, such as dysphagia and salivary disorders. These protocols must effectively address the challenges that these symptoms represent. In addition, qualitative research needs to be expanded to other neurological populations in order to enrich therapeutic strategies. In this way, treatments can be tailored to the individual experiences of patients, thereby improving their effectiveness and personalisation.

## Limitations

This study presents four main limitations. First, the geographical bias: since it was conducted exclusively in Spain with patients from a similar cultural background, the results may not be generalisable to other sociocultural contexts or healthcare systems. Second, the non-stratified sampling: the lack of specific quotas for motor stages (Hoehn & Yahr), Parkinson’s subtypes, or disease duration may affect the transferability of the findings. Third, the potential self-censorship in FGs: despite the use of experienced moderators, the group dynamics may have inhibited the expression of particularly sensitive experiences, such as feelings of embarrassment related to drooling or choking in public. Finally, the exploratory design: as a pioneering qualitative study on NMES for oropharyngeal symptoms in PD, the findings require validation through quantitative studies that enable broader generalisation.

## Conclusions

The results of this study offer valuable insights that can guide future research in the field of rehabilitation. They highlight critical aspects of treatment that cannot be fully explored through other methodologies. By incorporating the lived experiences of patients and their caregivers, we are better able to offer a more holistic and individualised approach to treatment, ultimately improving patient care and outcomes.

As the first qualitative study on NMES for oropharyngeal symptoms in PD, our major contribution is not simply to list the benefits but to reveal what aspects of clinical improvement actually impact daily life from the perspective of those who experience it. This knowledge is essential for designing rehabilitation approaches that patients perceive as meaningful.

## Data Availability

The datasets used and/or analysed during the current study are available from the corresponding author on reasonable request.

## Abbreviations

IC: Informed consent
OD: Oropharyngeal dysphagia
P: Patient
C: Caregiver
PD: Parkinson’s disease
NMES: Neuromuscular electrostimulation
QoL: Quality of life
HRQoL: Health-related quality of life
FG: Focus group
